# Isokinetic Knee Strengthening Impact on Physical and Functional Performance, Pain Tolerance, and Quality of Life in Overweight/Obese Women with Patellofemoral Pain Syndrome

**DOI:** 10.3390/jcm13164696

**Published:** 2024-08-10

**Authors:** Nadhir Hammami, Eya Bouzouraa, Cengiz Ölmez, Soukaina Hattabi, Najla Mhimdi, Mehrzia Amani Khezami, Pedro Forte, Andrew Sortwell, Anissa Bouassida, Monèm Jemni

**Affiliations:** 1 Research Unit (UR22JS01) “Sport Sciences, Health and Movement”, High Institute of Sport and Physical Education of Kef, University of Jendouba, Kef 7100, Tunisia; eya.bouzoraa@gmail.com (E.B.); hattabisoukaina@gmail.com (S.H.); mhimdinajla84@gmail.com (N.M.); bouassida_anissa@yahoo.fr (A.B.); 2 Physical Education and Sport Department, Sport Sciences Faculty, Ordu University, Ordu 52200, Türkiye; cengizolmez@odu.edu.tr; 3 Department of Physical and Rehabilitation Medicine, The National Institute of Orthopedics Mohamed KASSAB, La Manouba 2010, Tunisia; mehrziaamani.khezami@fmt.utm.tn; 4 Faculty of Medicine of Tunis, University of Tunis El Manar, Tunis 1007, Tunisia; 5 Department of Sports, Higher Institute of Educational Sciences of the Douro, 4560-708 Penafiel, Portugal; pedromiguel.forte@iscedouro.pt; 6 Research Center for Active Living and Wellbeing (Livewell), Instituto Politécnico de Bragança, 5300-253 Bragança, Portugal; 7 Research Center in Sports, Health and Human Development, 6201-001 Covilhã, Portugal; sortwellandrew@gmail.com; 8 Department of Sports, Instituto Politécnico de Bragança, 5300-253 Bragança, Portugal; 9 School of Health Sciences, University of Notre Dame Australia, Fremantle, WA 6959, Australia; 10 Department of Neurology, Carrick Institute, Cape Canaveral, FL 32920, USA; monemj@hotmail.com; 11 Faculty of Physical Education, Ningbo University, Ningbo 315211, China; 12 Centre for Mental Health Research in Association, University of Cambridge, Cambridge CB2 1TN, UK

**Keywords:** isokinetic dynamometer, strengthening, knee, patellofemoral syndrome, passive mode, obesity

## Abstract

**Background/Objectives:** Patellofemoral syndrome is a common osteoarticular condition that affects many individuals. Various treatment options are available, with a significant emphasis on targeted muscle-strengthening exercises. The purpose of this study was to investigate the effect of isokinetic muscle strengthening on muscle strength, joint range of motion, quality of life, physical performance, and pain tolerance in overweight/obese women with patellofemoral syndrome. **Methods:** Twenty-four overweight or obese women with patellofemoral syndrome participated in the study during September and October 2023 in a private medical facility for physical medicine and functional rehabilitation. They were randomly assigned to one of two groups for six weeks of isokinetic muscle strengthening. The first group (ISO.G) followed a rehabilitation program combined with isokinetic muscle strengthening. A second group (PCM.G) followed a rehabilitation program that includes an isokinetic protocol in passive compensation movement. The extensors’ peak torque was measured before and after training. **Results:** The flexors’ peak torque, stair climbing test, 10 m walk, chair lift, monopodal support, goniometric knee flexion test, heel–buttock distance measurement, pain, and quality of life scores improved significantly in both groups. The ISO.G, on the other hand, benefited from a significant increase in quadriceps muscle strength revealed by the extensors’ peak torque. **Conclusions:** For the treatment of patellofemoral syndrome, isokinetic muscle strengthening in concentric mode appears to have a significant advantage over the classic rehabilitation program with isokinetic passive compensation, particularly in muscle strength gain, in addition to the improvement of joint range of motion, quality of life, physical performance, and pain tolerance. Isokinetic training may be recommended as a beneficial approach for the rehabilitative treatment of patellofemoral pain syndrome in overweight/obese women.

## 1. Introduction

Over the last forty years, quality of life (QOL) has become a significant concept and area of research and practice in the health sciences and medicine [1]. Consequently, various instruments have been developed to measure QOL. One such instrument developed is the World Health Organization’s quality of life instrument, WHOQOL [2]. The WHOQOL collects subjective data on aspects of quality of life such as physical health, psychological health, social relationships, and environmental health. QOL is used to identify the range of problems that can affect patients. Health challenges or problems revealed by QOL instruments can be used to assist in developing plans for patients that lead to modifications and improvement in treatment and care [3].

Overweight and obese women with patellofemoral pain syndrome are often at greater risk of experiencing reduced QOL [4]. This is manifested through the resulting heightened pain, reduced ability to perform regular daily tasks, and limited mobility. The intersection of obesity and patellofemoral pain syndrome compounds current and future health challenges that adversely affect QOL by increasing susceptibility to chronic pain, emotional distress, and biomechanical impairments, thereby necessitating comprehensive management strategies to address the multifaceted impacts on their health and daily living [5].

Obesity and overweight rates have risen at an alarming rate around the world. Indeed, obesity is a major impedance to positive health outcomes, and it has been recognized as a disease since 1998 [6]. Obesity is a condition characterized by a person having a body mass index (BMI) of 30 or greater, calculated by dividing a person’s weight by the square of their height [7,8]. In comparison, the overweight classification is defined as a BMI of 25.0–29.9 [7,8]. Studies suggest that being overweight or obese poses a greater risk of death than being underweight [9]. Researchers have observed that overweight and different types of obesity, particularly abdominal obesity, are linked to a higher risk of various chronic and non-communicable diseases, including cancer; asthma, diabetes, high cholesterol, and cardiovascular diseases [10]. Obesity not only worsens existing conditions but also triggers new ones. Authors have also reported that obesity impacts nearly every organ system, including the cardiovascular, endocrine, central nervous, and gastrointestinal systems, and causes autoimmune diseases [11]. Moreover, obesity is associated with an increasing prevalence of several cardiovascular issues, such as hypertension, coronary heart disease, atrial fibrillation, and heart failure [10]. Damage in the musculoskeletal system is also observed [12,13,14]. Mechanical stresses on the cartilage of the large joints cause this damage [15]. Obesity and overweight place more strain on the patellofemoral joint than on other large mobile joints. Indeed, it is frequently subjected to loads of up to 11 times body weight. This is more noticeable during activities of daily living that require knee flexion, such as climbing/descending stairs, squatting, or running [16].

Patellofemoral syndrome (PFS) is a complex condition in terms of definition, etiology, evaluation, and treatment [17]. It is distinguished by pain in the anterior region of the knee [18,19]. The prevalence of this syndrome is 22/1000 cases per year and has a higher prevalence in women [20]. A number of risk factors can lead to PFS. The most common risk factor are increased quadriceps angle [21], decreased quadriceps and hamstring flexibility [22], and vastus medialis obliquus weakness [23].

This weakness is a very common symptom of muscle hypotrophy. The neuromuscular imbalance in the vastus medialis and vastus lateralis results in irregular lateral traction of the patella, which overloads the medial patellar retinaculum and the subchondral bone [24]. In addition to muscle strength, the vastus medialis may contract later than the vastus lateralis [25]. Nonetheless, a high body mass index (BMI) is not regarded as a risk factor for PFS [26]. Nevertheless, the increase in BMI can be explained by the abandonment of sports practice [18] due to the sensation of pain in the anterior knee region [27]. PFS can be treated, with research demonstrating that rehabilitation is an effective and important part of a prescribed treatment program. As a result, the prescription of specific quadriceps muscle strengthening and physical exercises produces positive outcomes [25,28]. Muscle strengthening exercises such as isokinetic dynamometry are recommended for the rehabilitation of PFS [29].

The popularity and acceptance of isokinetics as an effective training modality has grown in recent years among the scientific and research communities, specifically in muscle assessment, rehabilitation, and sports training [30,31,32,33,34]. Isokinetic dynamometers can control position, joint amplitude, movement speed, contraction mode, and exercise volume/intensity simultaneously. These benefits make them a valuable tool in rehabilitation, and their applications range from musculoskeletal pathologies [31,32] to neurological [35,36] and cardiovascular anomalies [37].

In the context of PFS, an isokinetic quadriceps strengthening program is recommended as part of the treatment strategy [38]. Nevertheless, there is a scarcity of evidence concerning the efficacy of this muscle strengthening technique as a rehabilitation regimen for non-athletic, overweight/obese women suffering from patellofemoral pain syndrome in terms of their functional capabilities, physical performance, pain threshold, and overall quality of life. Thus, the primary aim of this research was to investigate the impact of a concentric isokinetic knee strength-training program on muscle strength, joint range of motion, physical performance, quality of life, and pain perception in overweight/obese women with PFS. The hypothesis posited that integrating isokinetic muscle strengthening with traditional rehabilitation targeting the extensor–flexor muscles of the knee would help maintain and enhance strength, joint stability, and mobility. This combined approach was expected to lead to improved range of motion and increased pain tolerance in overweight/obese individuals with PFS.

## 2. Materials and Methods

### 2.1. Participants 

The patients were recruited from a private polyclinic’s physical medicine and functional rehabilitation department. To be eligible, participants must be female, between the ages of 30 and 50 years with a BMI greater than 25 kg/m^2^, suffering from anterior knee pain (bilateral), exhibiting quadriceps atrophy of at least five millimeters compared to the opposite limb, and diagnosed with patellofemoral pain syndrome (PFPS). Using weight grading scale criteria, the same physical therapist diagnosed both knees’ standard radiographs [39]. Patients who were pregnant, had a history of knee surgery, had resistance training, physical therapy, or any other type of rehabilitation therapy in the three months preceding the study, as well as patellar instability, cardiovascular disease, respiratory insufficiency, neurological diseases, diabetes managed with insulin, or cancer managed with chemotherapy were not included in the study. Moreover, patients were excluded if they withdrew from the study, missed two consecutive sessions in a week, sustained an injury, or expressed demotivation or lack of involvement in following the protocol. 

All potential participants for this research who met the inclusion criteria were informed of the research protocol and purpose of the research. Only after signed consent were they able to participate. This research study was conducted during 2 months, September and October 2023, and was wholly approved by the Scientific and Ethical Committees of the Research Unit (UR22JS01) of the High Institute of Sport and Physical Education of Kef, University of Jendouba, Tunisia (Code N°01-01-PRM-2023). The private polyclinic’s scientific, medical, and ethical committee accepted the study and the protocol was carried out in accordance of the Declaration of Helsinki.

The sample size calculation was conducted using G-Power software (G*Power 3.1.9.7). The analysis incorporated an effect size of f = 0.50, a power of 0.85, and a significance level of 0.05. It was determined that a minimum of 14 participants was necessary [40,41], leading to the selection of 24 participants who volunteered for the study and who also satisfied the specified criteria and completed the protocol. The benefits and risks of the experimental procedures used in the investigation were described and documented. Every subject has provided written consent to participate in the study. All participants gave their permission for their information to be used in this dissertation project and other scientific publications. 

**Figure 1:**
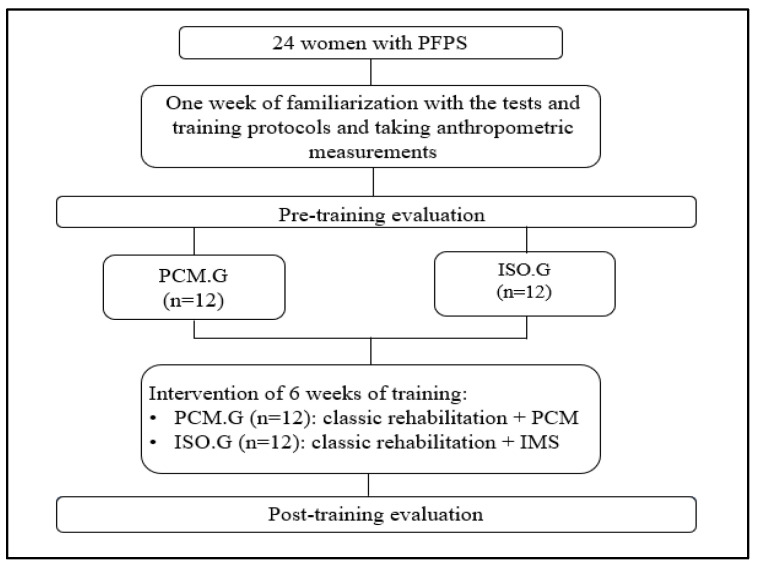
Flowchart of the study design.

Using a random number generator, study participants were divided into two groups based on the type of intervention used after the initial tests. A PCM group (PCM.G) completed a traditional rehabilitation program on the isokinetic dynamometer while working in Passive Compensation of Movement (PCM) mode. Meanwhile, an isokinetic group (ISO.G) executed the standard rehabilitation program plus an isokinetic muscle strengthening (IMS) program through maximum intensity (Figure 1).

Each group attended two sessions per week for six weeks [42]. The sessions were separated by at least one day. The number of repeats at 60°/s is shown in Table 1. There was a two-minute rest period between sets. The structured training program protocol in this study aligns with the program previously used by Alaca et al. [42].

### 2.2. Experimental Procedures

A week before the start of the program, seminars were held to familiarize participants with the evaluation processes and clinical testing. Participants were instructed to refrain from high-intensity activities for 24 h before the trial began. In addition, participants had to abstain from caffeine and other stimulant-containing beverages for the previous four hours. The program was performed at the same time and in the same location.

#### 2.2.1. Anthropometric Measurements

A systematic method was used to measure anthropometric characteristics (Table 2). Height was measured using a non-deformable and portable stadiometer (Seca model 213, Birmingham, UK) with the subject’s legs together, heels flat, neck and back straight, and head positioned in the sagittal axis [43]. The measurement is given in centimeters with a precision of 0.1 cm (cm). An impedance meter scale (OMRON BF 212, Kyoto, Japan) was used to calculate body weight, BMI, and fat percentage after entering the subject’s information (sex, age, and height) [31].

#### 2.2.2. Isokinetic Testing

The isokinetic muscle strength was measured using an ISOFORCE dynamometer (TUR GmbH, Berlin, Germany), which is a valid, reliable, and reproducible isokinetic machine [44]. Each patient’s joint axis was precisely aligned with the dynamometer’s axis of rotation. The subject was placed on the 90-degree adjustable seat, which was also adjusted for thigh length. The trunk, pelvic girdle, and thigh were held in place by stabilizing straps. The support lever was fastened between the upper and lower two-thirds of the leg. The subject was secured in place after adjusting the seat depth, dynamometer height, and inclination angle of the support lever. The axis of rotation of the dynamometer was aligned with an extended virtual axis of rotation for the knee, which was determined by a line through the femoral condyles. Following the adjustment for gravitational torque and the fine-tuning of the dynamometer mechanical stops to accommodate a range of motion of 80°, a targeted warm-up session was conducted in advance to acclimate participants to the physical exertion [45]. During a single test for each leg, the patient was asked to complete maximum knee flexion/extension in a concentric contraction mode at an angular velocity of 60°/s [33].

#### 2.2.3. Physical Fitness Testing

The stair climbing test (SCT), which timed how long it took them to ascend 11 regular stairs measuring 17 cm each, was one of four tests used to evaluate the participants’ level of physical performance [31,32,46]. The 10 m walk test determined how long it would take to cover 10 m at a leisurely pace [47]. The chair stand test measures lower extremity strength by simulating a squat [32,48]. The final test was the monopodal stance test, which determined how long it took to maintain balance [31,49].

#### 2.2.4. Functional Tests

Popliteal angular, knee range of motion, and heel-to-buttock distance were all measured during clinical evaluations.

The popliteal angle was assessed with the patient lying supine on the examination table, without any prior warm-up or stretching. The examiner flexed the hip to 90° and the knee to 90°, then instructed the patient to perform a passive knee extension to reach 0° inclination. The angle at which hamstring extensibility was limited was measured using a goniometer [50]. For the heel-to-buttock distance measurement, the patient was positioned prone, and the examiner passively flexed the knee. Using a tape measure, the distance between the heel and the gluteal groove was measured in centimeters [51] and the assessment was conducted for both legs. Finally, the range of motion of the knee joint was assessed utilizing a goniometer, positioned with its center on the lateral condyle of the knee. One arm of the goniometer was aligned along the axis of the femur towards the greater trochanter, while the other arm was aligned along the leg towards the lateral malleolus, all while the patient was lying supine [32,52].

#### 2.2.5. Evaluation of Knee-Related Quality of Life and Pain

In this study, a questionnaire called the “Questionnaire Knee Injury and Osteoarthritis Outcome Score (KOOS)” was used. This questionnaire is the most appropriate scale for PFPS patients due to its reliability, validity, and responsiveness [53]. It is a 42-item questionnaire that addresses five patient-specific dimensions: pain, other disease-specific symptoms, activities of daily living, sports and leisure, and knee-related quality of life [54]. In our study, we began with subscores for pain (KOOS-pain) and knee-related quality of life (KOOS-QoL).

### 2.3. Statistical Analysis 

The statistical analysis of the data was carried out using the “STATISTICA 10.0 (StatSoft. Inc.; Tulsa, OK, USA)” software for Windows. The results are presented as mean values less standard deviation. The Shapiro–Wilk test was applied to validate a study on the normality of all variable distributions in order to determine testing strategies (parametric or non-parametric). A repeated measures analysis of variance was conducted, with two components (training and group). For a paired comparison, the Bonferroni post hoc test was used (two to two). To examine the extent of the disparity between the variables, the eta squared (η*_p_*^2^) was analyzed to determine its magnitude. The significance of all statistical findings was determined using a probability level of less than 0.05.

## 3. Results

### 3.1. Muscle Strength 

The ANOVA analysis of the knee PTE differences between groups revealed (Table 3) that only the ISO.G benefited significantly from training (F = 10.453; *p* < 0.001; η_p_^2^ = 0.322; Δ% = 12.37). According to Bonferroni’s post hoc analysis, the muscular strength of the left and right knee extensors increased significantly after training. Furthermore, when Test and Re-Test values were compared, the ISO.G significantly improved (*p* < 0.001). In the knee PTF, the ANOVA revealed a significant training impact for the G.ISO (F = 19.592; *p* < 0.001; η_p_^2^ = 0.471; Δ% = 6.49) and a group training interaction (F = 10.89; *p* < 0.001; η_p_^2^ = 0.471; Δ% = 18.25) (Table 3). According to Bonferroni’s post hoc analysis, the strength of the left and right knee flexor muscles increased after training. Furthermore, when comparing Test and Re-Test values, there is a significant improvement in the ISO.G (*p* < 0.001).

### 3.2. Physical Performance

- SCT: There were no statistically significant group-by-training interactions found in the test before and after the program. Nonetheless, a significant interaction between Test and Re-Test was discovered in both groups (F = 280.562; *p* < 0.001; η_p_^2^ = 0.927; Δ% = 3.98). As a result, the Bonferroni post hoc test reveals a significant reduction in the time required to climb 11 stairs (*p* < 0.05). Furthermore, when comparing Test and Re-Test values, there is a significant improvement in the ISO.G and PCM.G (*p* < 0.001) (Table 4).
- 10 m Walk test: There was no discernible difference between groups after using the ANOVA test to compare pre- and post-training gait speed. However, both groups demonstrated a significant training effect (F = 193.897; *p* < 0.001; η_p_^2^ = 0.898; Δ% = 2.96). Furthermore, the Bonferroni post hoc test revealed a significant (*p* < 0.05) decrease in the time required to walk 10 m before and after training. As a result, the Test and Re-Test comparison of walking performance reveals a significant improvement in the ISO.G (*p* < 0.001) and PCM.G (*p* < 0.001) scores (Table 4).
- Chair stand test: When comparing the number of sit-ups performed before and after training for both groups, the ANOVA test shows a significant difference in training (F = 1331.365; *p* < 0.001; η_p_^2^ = 0.983; Δ% = 22.35) and no significant difference for group training (*p* < 0.05). Following that, the Bonferroni post hoc test reveals that the number of gesture repetitions requested during 30s is significantly higher after the protocol than before (*p* < 0.001) (Table 4).
- Monopodal stance test: When comparing the monopodal balance of both knees before and after the protocol, the ANOVA test revealed no discernible group training impact. The left knee (F = 167.150; *p* < 0.001; η_p_^2^ = 0.883; Δ% = 95.36) and the right knee (F = 396.068; *p* < 0.001; η_p_^2^ = 0.947; Δ% = 14.92) did, however, show a substantial training effect. When comparing the amount of time spent in the monopodal stance before and after the protocol, the Bonferroni post hoc test revealed a regression. The ISO.G and PCM.G of the right (*p* < 0.001) and left (*p* < 0.001) knees are thus confirmed to have improved significantly within the group (Table 4).

### 3.3. Clinical Examination

- Popliteal angle: The ANOVA test revealed a significant training effect not only in the right knee (F = 80.48; *p* < 0.001; η_p_^2^ = 0.785; Δ% = 30.56) but also in the left knee (F = 107.32; *p* < 0.001; η_p_^2^ = 0.829; Δ% = 37.22). However, the statistical test revealed no significant group training interaction in either knee (*p* < 0.05). Bonferroni’s post hoc test revealed a significant decrease in popliteal angle measured after the protocol compared to preintervention for both the right (*p* < 0.001) and left (*p* < 0.001) knees of both groups (Table 5).
- Knee ROM: in terms of flexion, Following the statistical ANOVA test, there was a significant effect of training for either the right (F = 80.45; *p* < 0.001; η_p_^2^ = 0.785; Δ% = 15.12) or left (F = 110.61; *p* < 0.001; η_p_^2^ = 0.834; Δ% = 12.35) knee. In contrast, no significant group training interaction (*p* < 0.05) was discovered. A Bonferroni post hoc test revealed that the flexion angle was greater post-protocol compared to the front for both knees at the group level (*p* < 0.001). In terms of extension, the ANOVA test shows that training has a significant effect on the right (F = 16.16; *p* < 0.001; η_p_^2^ = 0.423; Δ% = 20.83) and left (F = 16.08; *p* < 0.001; η_p_^2^ = 0.422; Δ% = 20.83) knees. However, group training had no significant effect (*p* < 0.05). Bonferroni’s post hoc test indicated that the angle of the extension was greater after training compared with the front for both knees of the CPM.G (*p* < 0.001) (Table 5).
- The heel-to-buttock measurement: The ANOVA test revealed that the comparison of heel-to-buttock distance in both groups before and after the protocol had no significant effect in the group training interaction (*p* < 0.05). However, there was a significant training effect for the right leg (F = 153.935; *p* < 0.001; η_p_^2^ = 0.874; Δ% = 18.61) and left leg (F = 252.485; *p* < 0.001; η_p_^2^ = 0.919; Δ% = 18.61). The Bonferroni post hoc test asserted that the heel-buttock distance is lower post-protocol compared to before and for both groups at both the right and left legs (*p* < 0.001) (Table 5).

### 3.4. Life Satisfaction and Pain

- QoL: The statistical analysis of variance (ANOVA) shows that there is no significant effect of group training interaction on quality of life (*p* < 0.05) but only a significant effect of the training protocol (F = 955.582; *p* < 0.001; η_p_^2^ = 0.977; Δ% = 86.86). According to the Bonferroni post hoc test, there was a significant decrease in the quality-of-life score after the protocol compared to before in the ISO.G (*p* < 0.001) and PCM.G (*p* < 0.001) (Table 6).
- Pain: According to the ANOVA study of variance, there was no significant difference for the group training effect. Nonetheless, a significant difference for the training effect was confirmed (F = 1109.40; *p* < 0.001; η_p_^2^ = 0.980; Δ% = 79.74). The Bonferroni post hoc test allows us to investigate this significance, and we discovered a significant pain decrease score after the protocol compared to before for the ISO.G (*p* < 0.001) and the PCM.G (*p* < 0.001) (Table 6).

## 4. Discussion

The goal of our study is to see how a knee concentric isokinetic muscle training program affects pain variation, physical qualities, and quality of life in overweight/obese women with PFPS. Our study focuses on non-operative approaches that include physical practice through muscle strengthening activities of the lower extremities generally and the quadriceps muscle as an important part of treatment [28,55,56,57]. Isokinetic measurements provide the most valid and reliable information for determining knee flexor and extensor strength in either the eccentric or concentric mode [58]. Concentric quadriceps and hamstring strength are lost during PFPS [59]. Concentric strength has been linked to a variety of actions, one of which is stair climbing [60]. This action heightens pain perception in the PF region [18,19]. As a result, concentric strength should be tracked to increase the prevalence of pain-free activity in the population of interest [60]. In this context, our previous findings demonstrate that isokinetic muscle strengthening improves muscle strength of the ISO.G flexors and extensors in concentric mode at a slow angular velocity of 60°/s. Hamdoun-Kahlaoui et al. [59] found that muscle strength increased after a concentric training program at various angular speeds (60°/s, 120°/s, and 180°/s). Werner and Eriksson [61] and Alaca et al. [42] confirmed that concentric training of the quadriceps produces significant results in strengthening this muscle at an angular speed of 60°/s, but they found no improvement in the hamstrings.

Furthermore, only the hamstrings of the left knee improved significantly in the PCM.G. The improvement in knee flexors could be attributed to the physiotherapist’s traditional rehabilitation. Our findings are consistent with the findings of van den Dolder and Roberts [62], who found that six sessions of manual therapy of the lateral aspect of the PF joint in patients with PFPS resulted in increased active knee flexion.

There is no significant intra-group interaction in our study. However, stair climbing, gait speed, chair lift, and monopodal support scores show a significant (*p* < 0.001) improvement for each group. According to studies, women with PFPS perform worse on some physical tests (chair stand test, stair-climb test, and 10 m walk test) than the healthy population [63]. Researchers argue that isokinetic muscle exercise is necessary for improving functional abilities [64]. This was also demonstrated by the study of Alaca et al. [42], which found a significant difference between all of the parameters measured in their functional evaluation. McMullen et al. [65] investigated the effect of the isokinetic program and the progressive static resistance and flexibility program on physical performance improvement. Following the program, the subjects, who were healthy people, were able to perform a variety of actions (walking, going down and upstairs). This study’s findings support those of the current study. The interpretation of the results obtained in this study reveals that both groups (ISO.G and PCM.G) benefited from their interventions, which allowed for a significant improvement in joint amplitude and muscle flexibility when compared to the pre-program evaluation.

It is evident that PFPS causes a variety of pathophysiological disorders, such as decreased joint range of motion and lack of flexibility in periarticular muscles [25], which appear to be determinants of physical disability [66]. A goniometric measurement of flexion, extension, popliteal angle, and heel–glute distance allowed us to assess the hamstring and quadriceps joint amplitude and flexibility [20]. To date, no study has looked at clinical examination results after an IMR program in a PFPS population. It is well established in the literature that joint range of motion and muscle flexibility are modifiable risk factors for PFPS [17,25].

As such, they are an essential component of the PFPS clinical examination. A loss of knee muscle flexibility can also significantly impact the biomechanics of the PF joint, increasing pain. Flexibility exercises for these muscles are advised in this case [17]. This explains our findings, which show significant improvements in joint range of motion and muscle flexibility following both the IMS program and traditional rehabilitation.

Several studies have found that patients in both groups experienced a significant decrease in pain, accompanied by improved quality of life [42,57,62,67,68]. According to these findings, IMS and traditional rehabilitation are effective at pain control [42,62]. In our case, the decreased level of physical activity is due to knee pain. As a result, people with PFPS avoid painful activities (such as squatting and stair climbing), resulting in functional disability [16].

According to the findings of this study, the positive effect of IMS on physical performance leads to an increase in pain tolerance. Furthermore, the IMS protocol and pain reduction improve knee-related quality of life and health [67].

In summary, this study encountered several limitations alongside the pertinent findings. In fact, certain patients experienced muscle soreness within the initial three weeks of the protocol, which proved intolerable, primarily attributable to insufficient recovery intervals between sets starting from the second week of training. This led to their contemplation of the substantial exertion demanded by the program. Furthermore, the research was confined to a female population afflicted with patellofemoral pain syndrome, thereby restricting the generalizability of the findings to broader populations with similar conditions. Moreover, the sequencing of knee evaluations during isokinetic procedures was non-randomized due to logistical considerations and patient convenience during experimental protocol evaluations. Lastly, the examiners’ assessments’ intra-rater reliability could not be established or quantified due to limitations in the time allocation for utilizing the experimental facilities and equipment.

## 5. Conclusions

The purpose of this study was to look into the effect of six weeks of isokinetic knee strength training on muscle strength, joint range of motion, physical performance, pain sensation, and quality of life in overweight/obese women with bilateral PFPS. 

We found that IMS shows an improvement in the muscle strength of the left and right knee flexors and extensors of the G.ISO in concentric mode at the angular velocity of 60°/s. Significant improvement was found only in the left knee hamstrings in the G.CPM. Furthermore, both groups benefited from their interventions, which resulted in significant improvement in joint range of motion and muscle flexibility compared to the assessment before the program. They also presented a significant reduction in pain led by an improvement in quality of life.

Our study highlights the effectiveness of the IMS program in concentric mode for improving muscle strength and flexibility, leading to enhancements in physical performance, joint range of motion, pain perception, and overall quality of life. Traditional rehabilitation interventions positively influenced some parameters and physical abilities but did not have the same effect on muscle strength. Therefore, we may recommend isokinetic training for the rehabilitative treatment of PFPS. These results are crucial for the management and treatment of PFPS, emphasizing the need to consider various training and rehabilitation modes for optimal outcomes.

## Figures and Tables

**Table 1:** Training program.

	Number of Rounds × Number of Repetitions		
	* Weeks 1 + 2	* Weeks 3 + 4	* Weeks 5 + 6
60°/s (Concentric)	3 × 5	3 × 7	3 × 10

*: 2 Sessions/Week.

**Table 2:** Anthropometric characteristics of the study population.

	ISO.G (*n* = 12)	PCM.G (*n* = 12)	*p*
Age (years)	53.50 ± 5.14	50.83 ± 9.50	0.4
Weight (Kg)	85.43 ± 10.96	87.33 ± 8.50	0.65
Height (cm)	1.58 ± 0.06	1.59 ± 0.08	0.67
BMI (kg/m^2^)	34.38 ± 4.59	34.72 ± 3.94	0.84
% Fat	41.44 ± 6.80	41.46 ± 9.21	0.99

*p*: statistical significance index; *n:* number. No significant differences were found between groups in all anthropometric characteristics (*p* < 0.05).

**Table 3:** PT of knee extensors and flexors recorded in concentric mode at 60°/s.

		Right Member		Left Member	
		PTE (Nm)	PTE (Nm)	PTE (Nm)	PTE (Nm)
ISO.G	Test	1.07 ± 0.36	0.52 ± 0.12	0.79 ± 0.25	0.42 ± 0.12
	Re-Test	1.41 ± 0.42 *	0.77 ± 0.16 *+	1.05 ± 0.21 *	0.39 ± 0.13 *+
PCM.G	Test	1.06 ± 0.46	0.52 ± 0.18	1 ± 0.40	0.66 ± 0.16
	Re-Test	1.03 ± 0.39	0.52 ± 0.22 *	1 ± 0.34	0.38 ± 0.11

Data are reported as mean ± standard deviation. ISO.G: Isokinetic Group; PCM.G: Passive Compensation of Movement Group; PTE: Peak Torque of Extensors; PTF: Peak Torque of Flexors; *: *p* > 0.001 Test vs. Re-Test; +: *p* > 0.001 ISO.G vs. PCM.G.

**Table 4:** Physical performance before and after the protocol.

		SCT (s)	10 m Walk Test (s)	Chair Stand Test (A.U.)	Monopodal Stance Test (s)	
					Right	Left
ISO.G	Test	7.41 ± 1.19	7.45 ± 0.82	10.08 ± 1.44	14.29 ± 5.69	15.66 ± 8.33
	Re-Test	5.21 ± 1.04 *	5.72 ± 0.40 *	18.83 ± 1.11 *	43.92 ± 6.74 *	50.59 ± 14.09 *
PCM.G	Test	7.48 ± 0.73	7.63 ± 0.70	9.08 ± 1.00	11.25 ± 4.80	12.38 ± 6.69
	Re-Test	4.95 ± 0.97 *	5.49 ± 0.50 *	19.17 ± 1.27 *	41.16 ± 11.02 *	50.05 ± 9.85 *

ISO.G: Isokinetic Group; PCM.G: Passive Compensation of movement Group; *: *p* < 0.001 Test vs. Re-Test; A.U.: Arbitrary Unit.

**Table 5:** Effect of different interventions on quality of life and pain.

		Popliteal Angle (Degrees)		Distance Heel to Buttocks (cm)		Flexion (Degrees)		Extension (Degrees)	
		Right	Left	Right	Left	Right	Left	Right	Left
ISO.G	Test	13.33 ± 8.35	13.75 ± 9.32	36.75 ± 6.12	36.33 ± 6.12	120.0 ± 11.28	122.08 ± 8.38	3.75 ± 6.44	3.75 ± 6.44
	Re-Test	7.92 ± 5.82 *	7.08 ± 5.42 *	29.92 ± 5.63 *	29.50 ± 4.96 *	137.0 ± 3.96 *	136.67 ± 3.26 *	1.67 ± 3.26	1.67 ± 3.26
PCM.G	Test	14.58 ± 5.42	15.83 ± 4.69	38.00 ± 4.65	38.50 ± 3.92	122.92 ± 6.20	123.75 ± 6.44	7.08 ± 7.22	7.08 ± 7.22
	Re-Test	5.83 ± 4.17 *	6.67 ± 3.89 *	28.08 ± 2.68 *	28.83 ± 4.09 *	135.00 ± 4.77 *	134.17 ± 6.34 *	3.75 ± 4.33 *	3.33 ± 4.44 *

ISO.G: Isokinetic Group; PCM.G: Passive Compensation of Movement Group; *: *p* < 0.001 Test vs. Re-Test.

**Table 6:** Clinical examination before and after training.

	Quality of Life (A.U.)		Pain (A.U)	
	Test	Re-Test	Test	Re-Test
ISO.G	29.75 ± 2.70	4.33 ± 1.92 *	29.75 ± 2.70	4.33 ± 1.92 *
PCM.G	26.33 ± 4.83	3.08 ± 1.88 *	26.33 ± 4.83	3.08 ± 1.88 *

ISO.G: Isokinetic Group; PCM.G: Passive Compensation of Movement Group; *: *p* < 0.001 Test vs. Re-Test; A.U.: Arbitrary Unit.

## Data Availability

Data is unavailable due to privacy and ethical restrictions which was agreed upon by both research parties.
